# Wafer-Level-Based Open-Circuit Sensitivity Model from Theoretical ALEM and Empirical OSCM Parameters for a Capacitive MEMS Acoustic Sensor

**DOI:** 10.3390/s19030488

**Published:** 2019-01-25

**Authors:** Jaewoo Lee, Jong-Pil Im, Jeong-Hun Kim, Sol-Yee Lim, Seung-Eon Moon

**Affiliations:** ICT Materials & Components Research Lab., Electronics and Telecommunication Research Institute (ETRI), Daejeon 34129, Korea; jpim@etri.re.kr (J.-P.I.); JeongHun@etri.re.kr (J.-H.K.); solyeeim@etri.re.kr (S.-Y.L.); semoon@etri.re.kr (S.-E.M.)

**Keywords:** open-circuit sensitivity, capacitive MEMS acoustic sensor, on wafer level, equivalent circuit-based modelling

## Abstract

We present a simple, accurate open-circuit sensitivity model based on both analytically calculated lumped and empirically extracted lumped-parameters that enables a capacitive acoustic sensor to be efficiently characterized in the frequency domain at the wafer level. Our mixed model is mainly composed of two key strategies: the approximately linearized electric-field method (ALEM) and the open- and short-calibration method (OSCM). Analytical ALEM can separate the intrinsic capacitance from the capacitance of the acoustic sensor itself, while empirical OSCM, on the basis of one additional test sample excluding the membrane, can extract the capacitance value of the active part from the entire sensor chip. FEM simulation verified the validity of the model within an error range of 2% in the unit cell. Dynamic open-circuit sensitivity is modelled from lumped parameters based on the equivalent electrical circuit, leading to a modelled resonance frequency under a bias condition. Thus, eliminating a complex read-out integrated circuit (ROIC) integration process, this mixed model not only simplifies the characterization process, but also improves the accuracy of the sensitivity because it considers the fringing field effect between the diaphragm and each etching hole in the back plate.

## 1. Introduction

Recently, micro-electromechanical systems (MEMS) microphones have been technologically competitive based on the maturity of mass production technology. Most commercial MEMS acoustic sensors in IoT applications are capacitive-type devices due to their compatibility with the standard complementary metal–oxide–semiconductor (CMOS) process in terms of form factor, thermal stability, and linear frequency response. The capacitive-type MEMS acoustic sensor [1,2,3,4,5,6,7,8,9,10,11,12,13,14,15,16] is composed of two plates, namely, a rigid back plate fixed on a substrate and a flexible diaphragm that detects an input acoustic signal. The moving plate should respond flexibly to the input sound pressure, and the hard bottom plate must have etch holes to reduce the damping effect. The characteristics of capacitive-type acoustic sensors are usually evaluated in terms of open-circuit sensitivity [1,3,9,11,14,15], which is determined by lumped parameters: the plate area of the capacitor, the spring constant of the flexible plate, the spacing of the two plates, and the ratio of the parasitic and intrinsic capacitance components. Although the open-circuit sensitivity at the static state can be roughly evaluated by pull-in voltage [1,4,9,11,15] from the capacitance change according to the applied voltage, it is difficult to obtain the parasitic capacitance considering the fringe field effect [17,18] due to the etching hole of the fixed electrode. Typically, the non-linear electric field of the etch hole makes it difficult to extract the fringe capacitance. As a result, the lateral capacitance model acting as such a parasitic component tends to be too simplistic, causing the electromechanical behavior to deviate from the proper condition. In addition, to investigate the frequency response, the first resonant frequency representing a linear frequency range must be modeled. Accordingly, for the measurement of the first resonant frequency, it is necessary to integrate the sensor with an ROIC converting a proper electric signal [3,5,7,8,9,11], which results in an intricate task. Considering the effective feedback, researchers developing such a sensor need an evaluation method that does not use an ROIC in the development stage, especially at the wafer level. Although the method of evaluating the open circuit sensitivity based on the equivalent circuit at the wafer level was introduced in [19], the parasitic components of the pad and the active region were not properly reflected. If the intrinsic capacitance in a capacitive-type acoustic sensor can be clearly modeled at the wafer level, the open-circuit sensitivity can be assessed without an ROIC connection in the frequency domain. However, such studies have not been investigated in depth because of the complexity of parasitic capacitance modeling.

In this paper, we present an efficient dynamic open-circuit sensitivity model mainly composed of both analytical ALEM-based [9,15] and empirical OSCM-based parameters [19] at the wafer level for a capacitive MEMS acoustic sensor. First of all, the overall structure of a capacitive acoustic sensor is described as a unit cell concept. Next, OSCM-based modeling is performed for the intrinsic capacitance of the active area of the capacitive acoustic sensor, where an additional sample without a membrane is used. Also, theoretical ALEM-based modeling is implemented to extract the parameters for the static open-circuit sensitivity evaluation. Subsequently, the validity of the proposed model is verified through finite element method (FEM) simulation. Next, the modeling sequence to investigate the dynamic response is described by using a modeling flowchart. Then, the process of extracting the static open-circuit sensitivity is presented. Finally, the dynamic open-circuit sensitivity is evaluated from both theoretical and empirical parameters.

## 2. On-Wafer Level Based Open-Circuit Sensitivity Model

In order to evaluate open-circuit sensitivity at the on-wafer level, we first examine the structure of a capacitive MEMS acoustic sensor and analyze the components of the required model to match the structure.

### 2.1. Capacitive MEMS Acoustic Sensors and Its Structure

**Figure 1:**
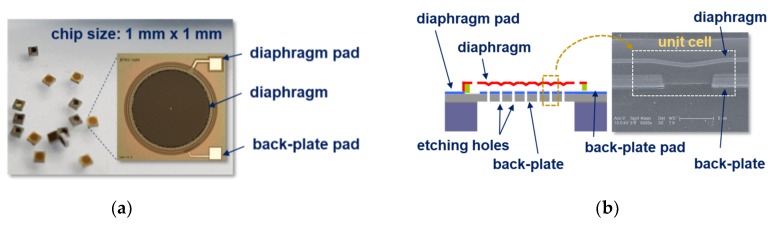
Schematic views and images of a capacitive MEMS acoustic sensor fabricated with a TiN/Al/TiN diaphragm: (**a**) top view; (**b**) cross section diagram and a SEM image.

A capacitive MEMS acoustic sensor operates on the principle of capacitance variation interacted with the pressure of the incident acoustic wave. A flexible diaphragm detecting an input acoustic signal allows the sensor to be specific to a capacitive senor. Considering the sensitivity requirements for IoT, the diaphragm has a diameter of 500 μm to 1000 μm and a thickness of approximately a few micrometers. The fixed electrode used as the lower electrode is arranged at air gap of 20 to 50% of the total area of the uniformly arranged etching holes in order to maintain the dynamic reaction of the diaphragm. Here, in general, the fixed electrode and the diaphragm are maintained at a few µm in air space. Figure 1a shows the MEMS acoustic sensor with the area of 1 mm × 1 mm [9], and Figure 1b describes the cross-sectional structure for a capacitor and the concept of a unit cell, where the SEM image shows the etched hole and the membrane fabricated on top of it with a certain space. The bottom electrode with etching holes was made of a SiO_2_/Si_3_N_4_ insulator/Al metal/a SiO_2_ insulator and the top was composed of TiN/PECVD-Si_3_N_4_/TiN multi layers. Air gap between the fixed electrode and the moving electrode was implemented by forming a polyimide sacrificial layer between the two electrodes and then removing it by O_2_ etching method.

### 2.2. Empirical OSCM Model

**Figure 2:**
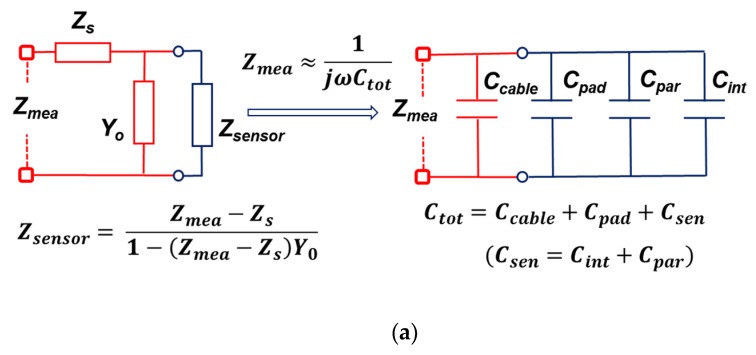

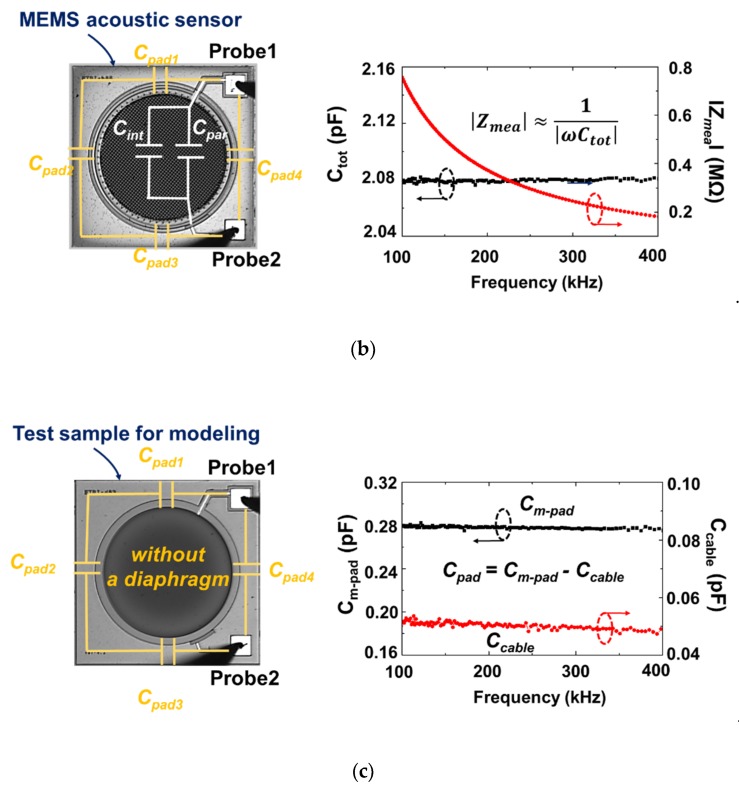
(**a**) The original circuit including the probe effect and the approximately circuit only consisting of capacitances; (**b**) structure-based circuit of capacitance for a capacitive MEMS acoustic sensor and its impedance measurement results; (**c**) structure-based circuit of capacitance for a sensor chip only containing the pad area and its impedance measurement results.

A capacitive acoustic sensor chip comprises an active area and a pad area. The active area is a part that reacts to external sound waves, and the pad area is a part where a negative pressure inputted by the acoustic sensor is converted into an electrical signal and the pad connects the signal to the lead-out circuit. The pad area on the whole chip is parasitic and can be extracted with additional samples. As shown in Figure 2a, the measured impedance value (*Z_mea_*) is composed of the serial impedance (*Z_s_*) and the parallel admittance (*Y_o_*) of the probe itself and the impedance (*Z_sensor_*) of the entire sensor chip. If *Z_s_* is negligible and *Y_o_* is simply simplified to cable capacitance (*C_cable_*), then the entire sensor chip will have the capacitance of the active area and the capacitance of the pad area. If it can be distinguished, it can be represented by the right equivalent circuit in Figure 2a. Here, the capacitance of the active region is subdivided into an intrinsic component (*C_int_*) and a parasitic component (*C_par_*). As in Figure 2b, the concentration parameters that make up the entire sensor chip can be configured. The measured total capacitance (*C_tot_*) was 2.09 pF. Also, the magnitude of the measured impedance (|Z_mea_|) was 0.21 MΩ at 360 kHz. To extract the capacitance of the active area, a membrane-less acoustic sensor chip was fabricated and the capacitance measured, as shown in Figure 2c. The measured cable capacitance (*C_cable_*) was 0.05 pF and the measured pad capacitance (*C_m-pad_*) was 0.28 pF, leading to the pad capacitance (*C_pad_*) of 0.23 pF. Thus, the active area capacitance (*C_sen_*) of the fabricated acoustic sensor was 1.81 pF under a bias condition of 0 V. It is clear that the capacitance value of the pad region can only be determined by extraction because it is an experimentally determined value.

### 2.3. Analytical ALEM Model

**Figure 3:**
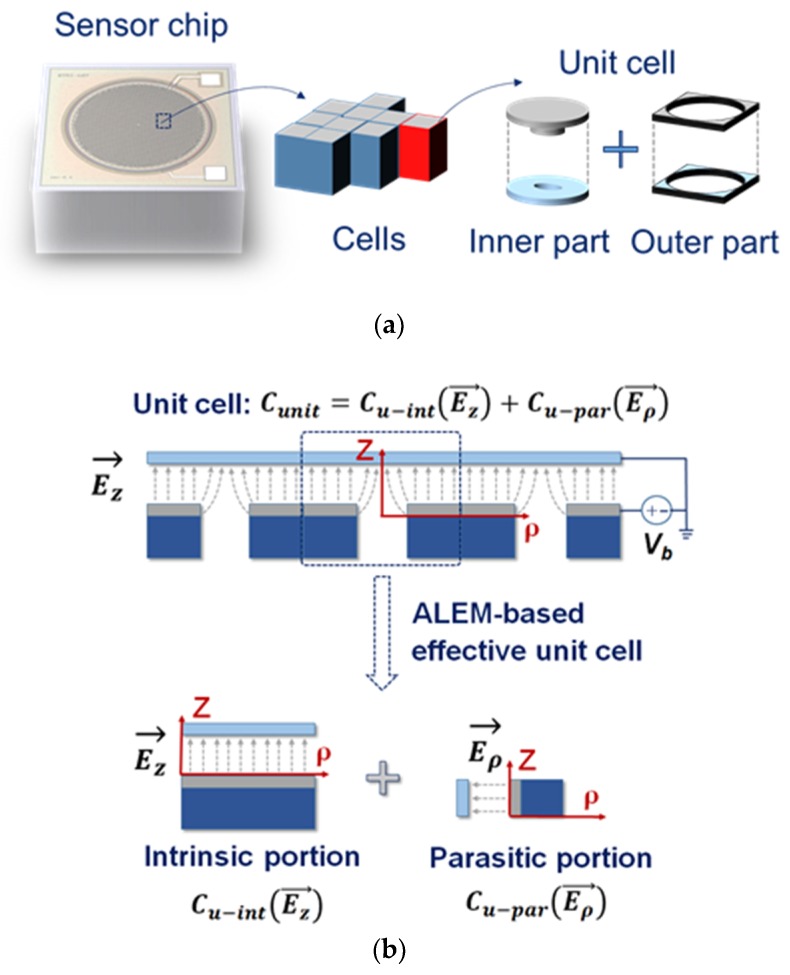
(**a**) Diagram of a capacitive MEMS acoustic sensor composed of unit cells; (**b**) diagram for the capacitive acoustic sensor roughly divided into an intrinsic and a parasitic component by the ALEM model.

As shown in Figure 3a, a capacitor of a MEMS acoustic sensor is composed of each unit cell, which was subsequently divided into two part: the inner and the outer part. The inner part is composed of a capacitor considering the fringe capacitance generated by the etching hole as a cylindrical coordinate axis, and the outer part is composed of a capacitance value with respect to the area of two equilibrium plates except the remaining part. Equation (1) shows the related *C_sen_* equation as

(1) $$C_{sen}=\frac{\oint \varepsilon_{0}\vec{E}\cdot d\vec{s}}{-\int_{g_{0}}^{0}\vec{E}\cdot d\vec{l}}=C_{int}\left(\vec{E_{z}}\right)+C_{fri}\left(\vec{E_{z}},\;\vec{E_{\rho }}\right),$$

where *ε*_0_ and *g*_0_ are the permittivity of vacuum and the height of air-gap. Here, it can be expressed as a distance with respect to the Z axis of the cylindrical coordinate axis. In addition, the electrostatic force (*F_ele_*) is the force acting on the opposing two electrode plates. The net force, which acts on the two plates placed perpendicular to the Z axis, is the force in the Z axis direction, as in Equation (2). Accordingly, the radial component becomes a parasitic component. In order to model the electrostatic force of the intrinsic component, the fringe capacitance generated by the etching hole should be divided in the axial direction and the radial direction. As shown in Figure 3b, the fringe capacitance can be linearized by the ALEM model [9,15]. In the unit cell, the z-axis direction (axial) becomes an intrinsic component and the radial direction becomes a parasitic component. *F_ele_* can be obtain as

(2) $$F_{ele}=\frac{\partial }{\partial z}\left(\frac{1}{2}C_{\mathrm{sen}}V_{b}{}^{2}\right)$$

(3) $$=\frac{V_{b}{}^{2}}{2}\frac{\partial }{\partial z}\left[C_{int}\left(\vec{E_{z}}\right)+C_{fri}\left(\vec{E_{z-f}},\;\vec{E_{\rho -f}}\right)\right]$$

(4) $$\approx \frac{V_{b}{}^{2}}{2}\frac{\partial }{\partial z}\left[C_{int}\left(\vec{E_{z}}\right)+C_{fri}\left(\vec{E_{z-f}}\right)\right],$$

where $C_{int}=C_{int}\left(\vec{E_{z}}\right)+C_{fri}\left(\vec{E_{z-f}}\right)$ and $C_{par}=C_{fri}\left(\vec{E_{\rho -f}}\right)$. *C_sen_* is the sum of the intrinsic and parasitic components of the active region and *V_b_* is the DC bias voltage applied to the capacitor. Theoretically, it is very complicated to model the fringe capacitance due to the structural features, but using the ALEM model is advantageous for extracting parasitic components.

### 2.4. Model Verification

**Figure 4:**
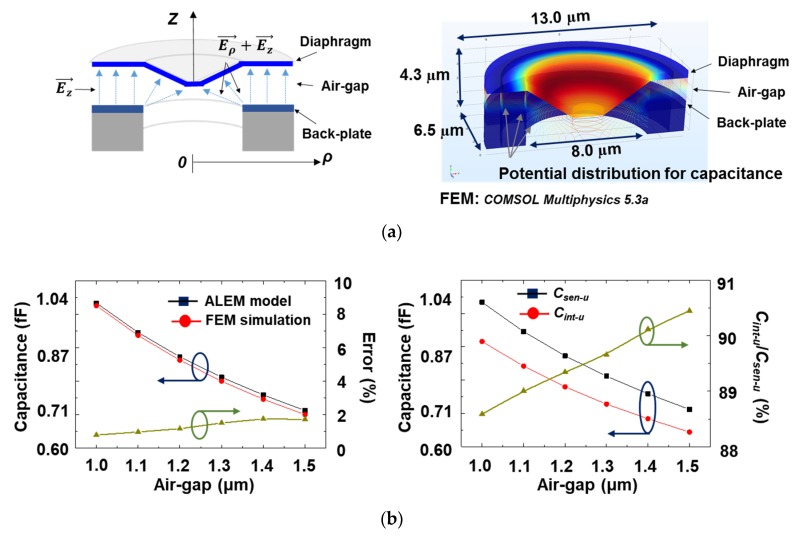
(**a**) Electric field distribution concept diagram of the inner part composing a unit cell and potential distribution by FEM simulation; (**b**) ALEM model and FEM simulation result and error result for the air gap-capacitance relation, and the total and the intrinsic capacitance for the air gap–capacitance relationship and its attenuation coefficient results.

To verify the analytical ALEM-based model, an FEM simulation was performed using a commercial simulator (*COMSOL Multiphysics 5.3 a*). Figure 4a shows the conceptual diagram of the capacitive MEMS acoustic sensor unit cell and the simulated electric field distribution. At electrode spacing from 1.0 to 1.5 μm, the modeled capacitance was in good agreement with the FEM simulation, with a unit cell radius of 6.5 μm and an etch hole radius of 4.0 μm. The modeled capacitance was 0.71 fF at 1.5 μm and 1.04 fF at 1.0 μm gap, which was the deviation of 1.7% at the 1.5 μm electrode spacing and 0.8% at the 1.0 μm electrode spacing compared to those of the FEM simulation. This analytical model proved its validity because it has error within 2% when compared with FEM simulation result.

In addition, the attenuation coefficient (*C_int-u_*/*C_sen-u_*) representing the ratio of the intrinsic component among the total capacitances was investigated in the modeled unit cell. As shown in Figure 4b, the attenuation coefficient was 90.4% at 1.5 μm electrode spacing and 88.6% at 1.0 μm spacing. These results show that the intrinsic component ratio is reduced by about 10% in the entire active region capacitance due to the etching holes. Therefore, the accurate attenuation factor must be properly reflected for open-circuit sensitivity extraction.

## 3. Dynamic Open-Circuit Sensitivity Modeling on Wafer Level

### 3.1. Modeling Process

**Figure 5:**
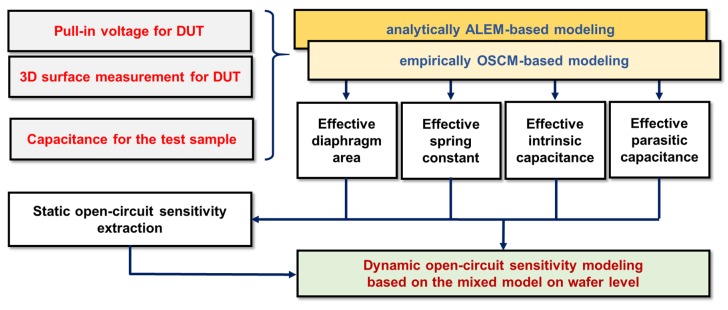
Flowchart for modeling dynamic open circuit sensitivity at the wafer level of capacitive MEMS acoustic sensors based on the ALEM and OSCM models.

The modeling flow chart is illustrated in Figure 5. First, we measure the C-V of the device under test (DUT) with an impedance analyzer to obtain the total capacitance and pull-in voltage. Then, the height of the membrane with respect to the fixed electrode of the DUT is measured by using a surface analyzer. Next, the capacitance of the test sample is extracted by an impedance analyzer to extract the parasitic capacitance of the pad region. When the measurement is complete, we extract the intrinsic component from the active area with the ALEM model. Also, the capacitance value of the pad region is extracted with the OSCM model. Next, we model the effective diaphragm area and effective residual stress when the capacitance value of the entire sensor chip is extracted with two models. In addition, we model the static open-circuit sensitivity with the modeled effective parameters. The attenuation coefficient and electrode spacing can be calculated using the ALEM model. Finally, we obtain dynamic open-circuit sensitivity with empirical or theoretical concentration variables that make up the electrical equivalent circuit. Here, the conversion area can be obtained through the modeled static open-circuit sensitivity.

### 3.2. Static Characterization

The static open-circuit sensitivity (*S_O_*) is evaluated by the bias voltage (*V_b_*), the air gap (*g_b_*) at a bias, the modelled diaphragm area (*A_mod_*), the modelled spring constant (*k_mod_*) and the damping constant (*C_int_*/*C_chi_*), where *C_chi_* = *C_int_* + *C_par_* + *C_pad_*. This can be expressed [1,3,9] by

(5) $$S_{o}=\frac{V_{b}}{g_{b}}\frac{A_{mod}}{k_{mod}}\left(\frac{C_{int}}{C_{int}+C_{par}+C_{pad}}\right).$$

**Figure 6:**
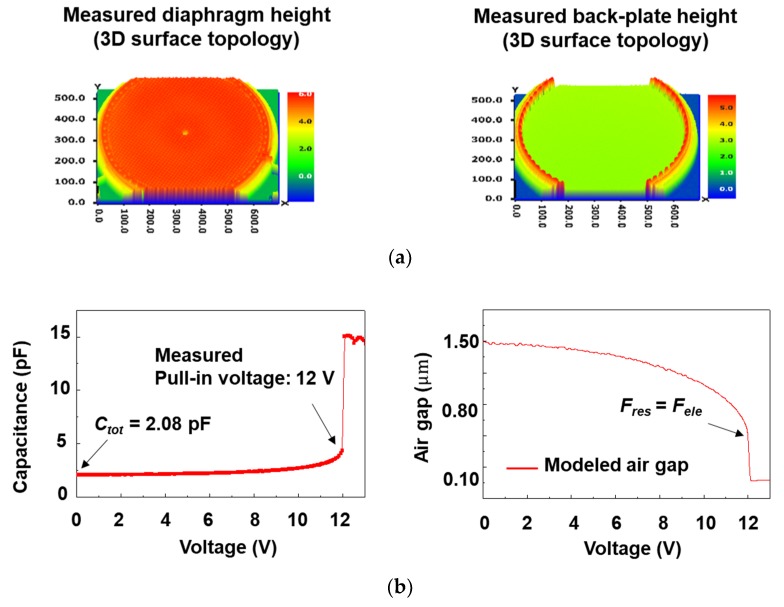

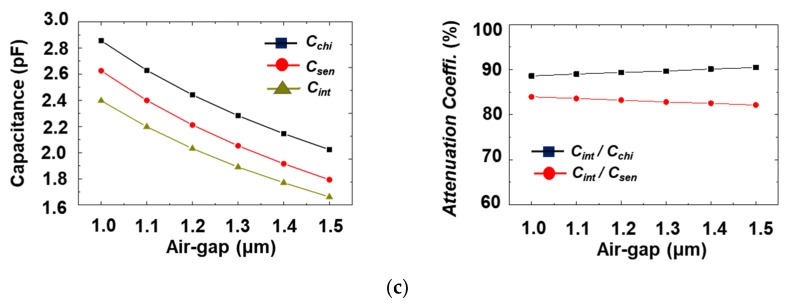
(**a**) Diaphragm and back-plate height of capacitive MEMS acoustic sensor measured by 3D surface analyzer; (**b**) the pull-in voltage according to the capacitance-voltage measurement and the air gap on the applied voltage of a modeled capacitive MEMS acoustic sensor; (**c**) modelled sensor chip capacitances, active area capacitances, intrinsic capacitance for the air gap and the intrinsic component ratio of the sensor chip to the air gap and the intrinsic component ratio of the active capacitance.

Figure 6a shows the diaphragm and back-plate height of capacitive MEMS acoustic sensor measured by 3D surface analyzer. The chip capacitance of the acoustic sensor can be extracted through pull-in voltage, 3D surface analyzer measurement, and OSCM model. Also, it is possible to determine the attenuation constant with the above two measurements and ALEM model. As shown in Figure 6b, the measured pull-in voltage was 12.0 V. The measured total capacitance (*C_tot_*) was 2.08 pF and the air gap was 1.5 μm at 0 V bias condition. In addition, the effective radius of 299 μm was extracted from the diaphragm radius of 325 μm for the first time through the ALEM model. Figure 6b is a graph showing the electrode gap versus the applied voltage for the modeling acoustic sensor as the effective radius. When a 12 V voltage was applied, a collapse occurred at 42% of the initial electrode spacing, leading to the collapse point (*g_col_*). Using the measured pull-in voltage, the effective spring constant of the diaphragm can be extracted. The point where the restoring force of the diaphragm is the same as the magnitude of the electrostatic force applied to the two positive plates is the collapse point. Therefore, the spring constant can be extracted by putting the restoring force and the electrostatic force inside the diaphragm into an equation. The restoring force (*F_res_*) acts perpendicularly on the diaphragm due to the small deflection of a diaphragm compared to its side-length, and it is given at a clamped circular diaphragm by [20,21]

(6) $$F_{res}\left(\vec{z}\right)=A_{mod}P_{res}\left(\vec{z}\right)=\left[4\pi \sigma_{mod}h+\frac{64\pi D}{\rho_{mod}{}^{2}}\right]g\left(\vec{z}\right)+\left[\frac{128\alpha \pi D}{h^{2}\rho_{mod}{}^{2}}\right]g{\left(\vec{z}\right)}^{3},$$

where *z* is the axis direction in a cylindrical coordinate, *h* is the diaphragm thickness, *σ_mod_* is the modelled residual stress of the diaphragm, *ρ_mod_* is the modelled radius of the capacitor, *D* is the flexural rigidity, and α is the empirical parameter dependent. On the other hand, assuming that the back-plate and the diaphragm are parallel, the electro-static force (*F_ele_*) can be represented by the differential of the effective capacitance in the nominal direction on the two plates from the electro-static energy. *F_ele_* can be represented [20,21] by

(7) $$F_{ele}\left(\vec{z}\right)=\frac{V_{b}{}^{2}}{2}\frac{\partial }{\partial z}\left[C_{int}\left(\vec{E_{z}}\right)+C_{fri}\left(\vec{E_{z-f}}\right)\right]=\frac{V_{b}{}^{2}}{2}\frac{\varepsilon_{0}\pi \rho_{mod}{}^{2}}{{\left[g_{0}-g\left(\vec{z}\right)\right]}^{2}}.$$

Also, if we configure the *F_ele_* equation as Taylor series [21], we will see the following result by

(8) $$F_{ele}\left(\vec{z}\right)=\frac{\varepsilon \pi \rho_{mod}{}^{2}V_{b}{}^{2}}{2g_{0}{}^{2}}+\frac{\varepsilon \pi \rho_{mod}{}^{2}V_{b}{}^{2}}{g_{0}{}^{3}}g\left(\vec{z}\right).$$

As a result, at pull-in equilibrium, *F_res_* is equal to *F_ele_* on the condition of *z* = *g_col_*. The extracted effective spring constant was 190 N/m. As shown in Figure 6c, the modeled capacitance (*C_sen_*) at the electrode gap of 1.0 μm was 2.63 pF and the intrinsic capacitance (*C_int_*) was 2.40 pF. The capacitance of the sensor chip (*C_chi_*) was 2.86 pF at the air gap of 1.0 μm, and the attenuation coefficient was decreased, resulting in 0.84. It was confirmed that the pad capacitance of 0.23 pF acts as a parasitic component with the attenuation coefficient reduced. The parasitic component of the pad should be minimized when designing the sensor. The static open-circuit sensitivity at 1.1 μm electrode spacing was modelled as 11.3 mV/Pa.

### 3.3. Dynamic Open-Circuit Sensitivity

**Figure 7:**
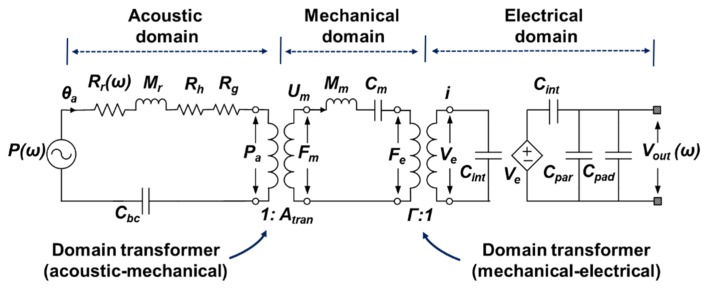
The electrical equivalent circuit consisting of three (acoustic-, mechanical-, and electrical-domain) regions for dynamic open-circuit sensitivity of a capacitive MEMS acoustic sensor.

An analogous electrical equivalent circuit composed of lumped parameters [11,15,22,23,24,25,26,27,28] was used to evaluate dynamic open- circuit sensitivity (*S_O_*) at the wafer level. Figure 7 shows an analogous electrical equivalent circuit for a capacitive MEMS acoustic sensor. It can be divided into three domains: the acoustic, the mechanical, and the electric domain. The conversion of each domain is divided by the transformer ratio. The effective conversion area in the acoustic-mechanical domain is the transformer ratio, while in the electro-mechanical domain, the effective charge per unit distance is the transformer ratio. Each lumped parameter can be easily extracted without using ROIC, which can be a useful tool to check the characteristics, especially at the sensor design stage. Dynamic *So* is defined to the ratio of the electrical output voltage (*V_out_*) to the input sound pressure (*P*) [11] as

(9) $$S_{0}\left(\omega \right)=\frac{v_{out}\left(\omega \right)}{P\left(\omega \right)}=\frac{A_{tran}}{A_{tran}{}^{2}X\left(\omega \right)+Y\left(\omega \right)}\cdot \frac{\Gamma }{j\omega C_{int}}\cdot \frac{C_{int}}{C_{int}+C_{par}+C_{pad}},$$

where *X(w)* and *Y(w)* are expressed by

(10) $$X\left(\omega \right)=R_{r}\left(\omega \right)+j\omega M_{r}+R_{h}+R_{g}+\frac{1}{j\omega C_{bc}},$$

(11) $$\left(\omega \right)=j\omega M_{m}+\frac{1}{j\omega C_{m}}+\frac{\Gamma^{2}}{j\omega C_{int}}.$$

Here, *θ_a_(ω)* is the volume velocity [m^3^/s], *M_r_* is the mass of the air close to the diaphragm (kg/m^4^), *R_r_(ω)* is the radiation resistance of the diaphragm (kg/s·m^4^), *R_g_* is the viscous resistance of the air-gap (kg/s·m^4^), *R_h_* is the viscous resistance of the acoustic holes (kg/s·m^4^), *C_bc_* is the compliance of the back chamber (m^3^/Pa), *P_a_(ω)* is the diaphragm portion of the input pressure that is converted to the related force in the mechanical domain (N/m^2^), *A_tran_* is the acoustic transduction factor (m^2^), *F_m_(ω)* is the force transformed to the mechanical domain (N), *U_m_*(ω) is the velocity (m/s), *M_m_* is the mass of the diaphragm (kg), *C_m_* is the compliance of the diaphragm (m/N), Fe(ω) is the intrinsic capacitance portion of the converted force (N), *V_e_*(ω) is the voltage transformed to the electrical domain (V), *Γ* the electrical transduction factor (C/m), *i*(ω) is the electrical current (A), and *V_out_*(ω) is the output voltage (V). In each domain, the main parameters are expressed by

(12) $$P\left(\omega \right)=\left(R_{r}\left(\omega \right)+j\omega M_{r}+R_{h}+R_{g}+\frac{1}{j\omega C_{bc}}\right)\theta_{a}\left(\omega \right)+\frac{F_{m}\left(\omega \right)}{A_{tran}},$$

(13) $$F_{m}\left(\omega \right)=\left(j\omega M_{m}+\frac{1}{j\omega C_{m}}\right)U_{m}\left(\omega \right)+\Gamma V_{e}\left(\omega \right),$$

(14) $$V_{e}\left(\omega \right)=-\frac{i\left(\omega \right)}{j\omega C_{int}}=\frac{\Gamma }{j\omega C_{int}}U_{m}\left(\omega \right),$$

(15) $$V_{out}\left(\omega \right)=\frac{C_{int}}{C_{int}+C_{par}+C_{pad}}V_{e}\left(\omega \right).$$

As a result, for the dynamic *S_O_*, these equations should be linked and subtracted, leading to the transfer function of the output voltage as a function of the input pressure. Subsequently, in the acoustic domain, the mass of the air close to the diaphragm and the radiation resistance of the diaphragm are expressed [24] by

(16) $$R_{r}\left(\omega \right)=\frac{1}{2\pi }\frac{\rho_{0}\omega^{2}}{c},$$

(17) $$M_{r}=\frac{8}{3}\frac{\rho_{0}}{\pi^{2}}\frac{1}{\rho_{tran}},$$

where *ρ*_0_ is the density of the air, *ρ_tran_* is the transduction radius of the diaphragm, ω is the sound circular frequency, and c is the sound velocity. *R_r_* has a frequency-dependent value. *M_r_* was 322 kg/m^4^ at 10 V. The viscous resistance of the back-plate holes [2] is given by

(18) $$R_{h}=\frac{8\upsilon t_{b}}{N\pi r_{b}{}^{4}},$$

where *t_b_* is the thickness of the back-plate, *υ* is the viscosity of air, and *r_b_* is the radius of the back-plate etching hole. *R_h_* was 1.51 × 10^8^ kg/s·m^4^ at 10 V. As the air-gap can be considered as a purely resistive element, the viscous resistance of the air-gap [2], can be determined by

(19) $$R_{g}=\frac{12\upsilon }{N\pi g_{b}{}^{3}}\left(\frac{A_{p}}{2}-\frac{A_{p}{}^{2}}{8}-\frac{lnA_{p}}{4}-\frac{3}{8}\right),$$

where *N* is the hole number in the back-plate and *A_p_* is the surface fraction occupied by the acoustic holes. *R_g_* was determined to be 1.88 × 10^9^ kg/s·m^4^ at 10 V. It is obvious that *g_b_* acts as the critical parameter to determine *R_g_*. Also, *C_bc_* is considered as the air resistance of the chamber, which is given by [24]

(20) $$C_{bc}=\frac{V_{cham}}{\rho_{0}c^{2}},$$

where *V_cham_* is the volume of the back chamber. *C_bc_* was calculated to be 7.06 × 10^−14^ m^3^/Pa at 10 V.

**Figure 8:**
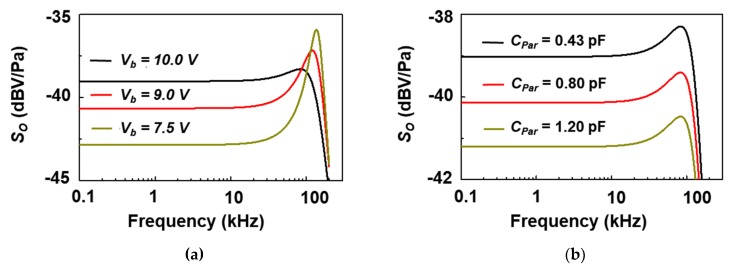
(**a**) Modelled dynamic open-circuit sensitivities based on bias voltages (7.5 V, 9.0 V, 10.0 V) of a capacitive MEMS acoustic sensor; (**b**) modelled dynamic open circuit sensitivities according to parasitic capacitances (0.43 pF, 0.80 pF, 1.20 pF) at 10.0 V bias condition of a capacitive MEMS acoustic sensor.

Moreover, in the mechanical domain, the equivalent mass of the diaphragm can be calculated by

(21) $$M_{m}=\rho_{d}h(A_{tran}/3),$$

where *ρ_d_* is the equivalent density of the diaphragm and the value of *A_tran_*/3 is calculated by considering the area as a lumped diaphragm. *M_m_* was modelled to be 2.52 × 10^−10^ kg at 10 V. The compliance of the diaphragm (*C_m_*) can be modeled by

(22) $$C_{m}=\frac{1}{k_{tran}},$$

where *k_tran_* is the transduction spring constant of the diaphragm, which is equal to *k_mod_*/3 due to the assumption of the lumped diaphragm. *C_m_* was modelled to be 1.58 × 10^−2^ m/N at 10 V. Figure 8a shows the modeled dynamic *S_O_* with bias conditions. The dynamic open-circuit sensitivities were −42.9 dBV/Pa, −40.6 dBV/Pa, and −39.0 dBV/Pa at 7.5 V, 9 V, and 10 V bias conditions, respectively. Also, the resonant frequencies (*f_r_*) were 136.1 kHz, 120.6 kHz, and 83.1 kHz, respectively, at 7.5 V, 9 V, and 10 V bias conditions. From these results, it is confirmed that the damping coefficient decreases as the electrode gap increases, but the sensitivity increases under the condition of the pull-in voltage. Figure 8b shows the effect of parasitic capacitance. The dynamic open circuit sensitivities were −39.0 dBV/Pa, −40.1 dBV/Pa, and −41.2 dBV/Pa, respectively, when the parasitic components were 0.43 pF, 0.80 pF and 1.2 pF at 10 V bias condition, reflecting a decrease in sensitivity. Since the parasitic component of 0.4 pF reduces the sensitivity of about 1 dB, it can be understood that the structure should be determined in the sensor design stage so that the parasitic component can be suppressed as much as possible. Table 1 shows the comparison data of this research with current models.

The modeling process is summarized as follows.

1. Determine *C_tot_* and *V_p_* by measuring the voltage-capacitance relation of the DUT with an impedance analyzer.
2. Determine the air gap (*g*_0_) by measuring the height of the diaphragm with a 3D surface analyzer.
3. Extract *C_pad_* of sensor chip with OSCM model.
4. Determine *C_int_* and *C_par_* of the active area with the ALEM model.
5. Model the spring constant (*k_mod_*) and the capacitor area (*A_mod_*) by extracting the voltage-electrode spacing relationship from the ALEM model and pull-in measurement results.
6. Obtain a static open-circuit sensitivity (*S_O_*) at an arbitrary air gap (*g_b_*).
7. To model the dynamic open-circuit sensitivity, we assume a distributed diaphragm as a lumped diaphragm, and determine the lumped parameters (*R_r_*, *M_r_*, *R_h_*, *R_g_*, *C_bc_*, *M_m_*, *C_m_*, *C_int_*, *C_par_*, and *C_pad_*). Owing to the lumped diaphragm, the area of *M_m_* is *A_tran_*/3 and the magnitude of the spring constant (*k_tran_*) in the dynamic range is *k_mod_*/3.
8. The acoustic transduction factor (*A_tran_*) is modeled as the determined static *S_O_* is equal to dynamic *S_O_* at the condition of ω = 0.
9. The electrical transduction factor (*Γ*) is determined as *C_int·_V_b_*/*g_b_*.
10. Finally, dynamic *S_O_* is modeled with extracted lumped parameters.

Thus, we have presented a dynamic open circuit sensitivity modeling method that takes into account all the parasitic components of a sensor chip on wafer level without using ROIC. Modelled and extracted parameters are given in Table 2. This modeling result proves that it can be used as a design consideration variable in the development stage of the sensor.

## 4. Conclusions

We demonstrated an electrical equivalent circuit-based dynamic open-circuit sensitivity model, which is based on both analytical ALEM-based and empirical OSCM-based parameters for a capacitive MEMS acoustic sensor at the wafer level. The ALEM-based model can be used to analyze the influence of intrinsic components, and the OSCM-based model can be used to analyze the parasitic components of the sensor chip. Since the fringe capacitance generated in the etching hole is included in the modeling process, the reliability of the model can be secured. Through FEM simulation, we verified the validity of the proposed model with an error range of less than 2%. This modeling approach based on these two methods offers the advantage of evaluating dynamic open-circuit sensitivity at the wafer level without the use of an ROIC. If evaluated at the wafer level, performance can be easily assessed without the conventional complex packaging process, which can be advantageous for researchers working on sensor development.

## Figures and Tables

**Table 1:** Comparisons between the proposed study and the currently published models.

	Model 1 [22]	Model 2 [24]	Model 3 [26]	This Work
Circuit model	3 domain-based circuit	Mixed domain-based circuit	Mixed domain-based circuit	3 domain circuit inserted with VCVS
Fringe field effect	Not mentioned	Not mentioned	Not mentioned	Included
Attenuation coefficient	Partially considered	Not considered	Partially considered	Fully considered
Evaluation	-	With ROIC	With ROIC	On wafer level
Features	Only proposed	Too simplified	Simplified	One test sample needed

**Table 2:** Modelled parameters and variables for a capacitive MEMS acoustic sensor.

Parameters and Characteristics	Values
Intrinsic capacitance (*C_int_*) at the bias of 10 V	2.26 pF
Parasitic capacitance (*C_par_*) at the bias of 10 V	0.20 pF
Pad capacitance (*C_pad_*)	0.23 pF
Air gap (*g_b_*) at the bias of 10 V	1.1 um
Modelled capacitor area (*A_mod_*)	2.81 × 10^−7^ m^2^
Modelled spring constant (*k_mod_*)	190 N/m
Acoustic transduction factor (*A_tran_*) at the bias of 10 V	3.69 × 10^−7^ m^2^
Electrical transduction factor (*Γ*) at the bias of 10 V	2.05 × 10^−5^ C/m
Dynamic open-circuit sensitivity (*S_o_*) at the bias 10 V	−39.0 dBV/Pa
First resonance frequency (*f_0_*) at the bias 10 V	83.1 kHz
